# Estimation of Selected Minerals in Aortic Aneurysms—Impaired Ratio of Zinc to Lead May Predispose?

**DOI:** 10.1007/s12011-020-02410-6

**Published:** 2020-10-01

**Authors:** Katarzyna Socha, Alicja Karwowska, Adam Kurianiuk, Renata Markiewicz-Żukowska, Andrzej Guzowski, Marek Gacko, Tomasz Hirnle, Maria H. Borawska

**Affiliations:** 1 Department of Bromatology, Medical University of Bialystok, Bialystok, Poland; 2 Department of Hygiene, Epidemiology and Metabolic Disorders, Medical University of Bialystok, Bialystok, Poland; 3 Department of Vascular Surgery and Transplantation, Medical University of Bialystok, Bialystok, Poland; 4 Department of Cardiosurgery, Medical University of Bialystok, Bialystok, Poland

**Keywords:** Aortic aneurysm, Copper, Zinc, Selenium, Cadmium, Lead

## Abstract

The objective of this study was to estimate the content of copper, zinc, selenium, cadmium, and lead in the tissue of patients with aortic aneurysms. Molar ratio of Cu/Zn and antioxidant micronutrients to toxic elements was also calculated. A total of 108 patients: 47 with abdominal aortic aneurysm (AAA), 61 patients with thoracic aortic aneurysm (TAA), and a control group of 20 abdominal aortic (AA) and 20 thoracic aortic (TA) wall samples from the deceased were studied. The concentrations of mineral components in the tissue samples were determined by the AAS method. The average concentration of Cu in the aortic wall of patients with TAA was significantly lower than in the aortic wall samples of healthy people. The mean concentration of Zn in the aortic wall of patients with AAA and TAA was significantly lower than in the control group samples. Cu/Zn ratio was significantly higher in AAA patients than in control group which indicates a greater role of oxidative stress and inflammatory process in this type of aneurysm. The concentration of Se was significantly decreased in TAA patients compared with the control group; in turn, the concentration of Pb was increased in this group of patients. We observed significantly lower Cu/Pb ratio in TAA patients than in control group, whereas Zn/Pb ratio was significantly lower comparing with control samples in both types of aneurysms. In the examined aneurysms, we have shown the differences in concentrations of mineral components compared with the control tissues. The Zn concentration was decreased in both AAA and TAA samples. Impaired ratio of Zn to Pb may predispose to aortic aneurysms.

## Introduction

An aneurysm is an excessive increase in the diameter of an artery caused by weakening of the arterial wall, resulting in an abnormally large bulge. Aneurysms may remain asymptomatic or rupture, causing serious health problems and even death [1]. The aorta is the largest blood vessel in the body and is a common site for arterial aneurysms. Aneurysms in the chest area are called thoracic aortic aneurysms (TAA). Abdominal aortic aneurysms (AAA) are the most common type. AAA is diagnosed in 1.4% of people aged between 50 and 84, 5.7 times more frequently in men than in women. The incidence of AAA increases with age [2]. In rare cases, damage to the arteries can affect both the chest and abdomen. The pathogenesis of aneurysm formation and rupture is not yet fully understood. Although the exact cause of an aneurysm is unclear, certain factors contribute to the condition. It is considered that damaged tissue in the arteries can play a role in the pathogenesis. Atherosclerotic disease, high blood pressure, inflammation, and infection can predispose to the formation of aneurysm. The vast majority of AAA is atherosclerotic. In contrast, aneurysms affecting the thoracic aorta (TA) are usually caused by medial degeneration which can occur during normal aging process, but may be accelerated by other conditions, such as hypertension, bicuspid aortic valve, and genetic changes. Recent advances in the genetic and molecular mechanisms underlying TAA demonstrated a strong genetic component, and several classes of genes responsible for TAA have been identified [3]. Certain dietary factors have an impact on the risk of developing an aneurysm, including high-cholesterol diet, fatty meat, alcohol consumption, inadequate intake of nuts, fruits, and vegetables. Smoking cigarettes also has a negative effect [2].

The role of copper (Cu) in the human body is connected to its participation in the structures and functions of many enzymes. The function of Cu in the inflammation process has not been exactly defined. However, the most important is the control of synthesis of free oxygen radicals, due to the presence of this element in Cu/Zn—superoxide dismutase. Cu plays an important role in the synthesis of collagen and elastin. The lysyl oxidase is a Cu-dependent extracellular enzyme which catalyzes formation of aldehydes from lysine residues in elastin and collagen precursors. The changes in the content of Cu in the arterial wall are important because deficiency of this mineral component may impair the formation of cross-links in elastin and collagen molecules and lead to the weakening of the resilient properties of elastin and mechanical resistance of collagen [4]. However, an excess Cu, with a simultaneous deficiency of zinc (Zn), can lead to oxidative stress and the formation of an inflammatory process [5]. Zn is one of the most important trace elements in the human body. This mineral component takes part in over 300 enzymatic reactions and is essential for DNA synthesis, RNA transcription, activation, and cell division. Zn plays an important role in wound healing, biosynthesis, and homeostasis of connective tissue [6, 7]. As a component of Cu/Zn, superoxide dismutase Zn has an antioxidant and anti-inflammatory activity obviously regulating pathogenesis of the inflammation-related diseases [8–10]. Zinc also plays a key role in regulating the function of gelatinases MMP-2 and MMP-9, the group of zinc-dependent matrix metalloproteinases (MMP), whose expression increases during inflammation with artery damage [11]. Selenium (Se) is a trace element necessary for the proper functioning of the body, but its excess also has negative effects. Se is a component of the active center of glutathione peroxidases (GPXs). Due to reaction with peroxides, Se prevents the body against harmful effects of free radicals, and therefore, it has antioxidant and anti-inflammatory properties [12, 13]. Se also has a detoxifying effect in the case of exposure to toxic elements, such as lead (Pb) and cadmium (Cd) [14]. Cadmium (Cd) and lead (Pb) are toxic elements, taken from drinking water and food. After absorption into the blood, Cd and Pb may accumulate in the liver, kidney, brain, and bone. Cd has a negative effect on the activity of oxidases and induces lipid peroxidation [15]. Chronic exposure to Cd can cause cardiovascular diseases. Cd accumulates in the liver and kidney in the form associated with metallothionein. It is considered that Cd can affect the development of hypertension, which is probably related to insufficient renin release from the hypoxic kidney, which accumulates large amounts of metallothionein [16]. One of the main toxic effects of Pb is inhibition of hemoglobin synthesis and a negative influence on the functioning of the circulatory system. With chronic exposure, kidneys and liver may also be damaged [17, 18]. Pb may induce oxidative stress and is considered as a potential immunotoxic factor [19]. Oxidative stress has been suggested to play a key role in the pathogenesis of AAA [20, 21]. However, only few studies have been conducted to evaluate the oxidative stress status of AAA patients. Some studies reported imbalance of mineral components in patients with aneurysms [22–25].

The objective of this study was to estimate the content of microelements: copper (Cu) zinc (Zn), selenium (Se) and toxic elements: cadmium (Cd), lead (Pb) in the tissue of patients with aneurysms. Molar ratio of Cu/Zn and antioxidant micronutrients to toxic elements was also assessed.

## Material and Methods

### Characteristic of the Examined Groups

A total of 108 patients: 47 with abdominal aortic aneurysm AAA (41 men and 6 women, aged 46–86, with a mean age of 69.7 ± 9.9 years) and 61 patients with thoracic aortic aneurysm TAA (41 men and 20 women, aged 22–83, with a mean age of 60.1 ± 13.6 years), and a control group of 20 abdominal aortic (AA) and thoracic aortic (TA) wall samples from the deceased (aged 21–68, with a mean age of 52.4 ± 14.5 years) were studied. Normal aortas were collected from people fatal casualties of traffic accidents, not suffering from chronic diseases such as diabetes and cardiovascular diseases. The size of aneurysm was assessed by ultrasound method. Eighty-seven percent AAA and 79% TAA patients smoked cigarettes (20–40 a day). Forty-five percent of patients with AAA and 15% patients with TAA declared drinking alcohol (vodka) more than once a week in amount 50–100 g. Patients and control group data are shown in Table 1.

**Table 1:** Characteristic of the examined groups

Variable	Control group	AAA patients	TAA patients
Total	20	47	61
Gender (M/F)	(10/10)	(41/6)	(41/20)
Age (years): average ± SD (range)	52.4 ± 14.5 (21–68)	69.7 ± 9.9 (46–86)	60.1 ± 13.6 (22–83)
Aneurysm size (mm) average ± SD (range)	-	73.815 ± 18.47 (50–120)	53.377 ± 8.56 (30–92)
Smoking cigarettes^a^/no-smoking	nd	41/6	48/13
Alcohol drinking^b^/no-drinking	nd	21/26	9/52

*AAA* abdominal aortic aneurysm, *TAA* thoracic aortic aneurysm, *SD* standard deviation, *nd* no data

^a^20–40 cigarettes/daily

^b^More than once a week

### Collection of Samples

Aneurysmal and control samples were taken during aneurysmectomy and autopsy, respectively, then were frozen at − 80 °C in the polypropylene tubes. The protocol of the study was approved by the Local Ethical Committee (R-I-002/401/2013).

### Sample Preparation and Determination of Mineral Components

Samples weighing approximately 0.3 g (with the accuracy of 1 mg) were digested in the concentrated nitric acid using the microwave mineralizer (Berghof, Germany). After the procedure, the samples were quantitatively transferred to polypropylene vessels (20-fold dilution) with ultrapure water. The concentrations of mineral components in the tissue samples were determined by the flame (Cu, Zn) and the electrothermal (Se, Cd, Pb) atomic absorption spectrometry method with the Zeeman background correction (Z-2000 instrument, Hitachi, Japan). The assessments were performed in triplicate. Certified reference material–bovine muscle BCR 184 (Commission of the European Communities, Community Bureau of Reference BCR, Brussels) was used to test the accuracy of the methods. The results of the quality control analyses corresponded with the reference values. The recovery for analytical methods used in estimation of Cu, Zn, Se, Cd, and Pb was 101%, 98.5%, 97.5%, 103%, and 102.5%, respectively. The Department of Bromatology, Medical University of Bialystok participates in a quality control program for trace element analysis supervised by the National Institute of Public Health–National Institute of Hygiene and the Institute of Nuclear Chemistry and Physics. Molar ratio of Cu/Zn and antioxidant micronutrients to toxic elements was calculated using Microsoft Excel software.

### Statistical Analysis

Statistical analyses were performed using Statistica v.12.0 software. Data were tested for normal distribution by the Kolmogorov-Smirnov and the Shapiro-Wilk tests. Differences between independent groups were tested by the nonparametric Mann-Whitney *U* test. Correlation was calculated and tested by Spearman rank test.

## Results

### The Concentration of Antioxidant Microelements

The average concentration of Cu in the aortic wall of patients with TAA was 1.04 ± 0.32 μg/g and was significantly lower (*p* < 0.02) than the content of Cu in the aortic wall samples of healthy people: 1.19 ± 0.25 μg/g. In addition, smokers had a significantly lower (*p* < 0.04) concentration of Cu in the aneurysmal tissue, compared with non-smokers, 0.985 ± 0.22 μg/g vs. 1.314 ± 0.52 μg/g. Data are not shown in Table 2. The average content of Cu in the arterial wall of patients with AAA was 2.15 ± 1.39 μg/g and did not significantly differ (*p* = 0.773) from the concentration of Cu in arterial wall samples of healthy people: 1.75 ± 0.69 μg/g.

**Table 2:** The content of zinc, copper, selenium, cadmium, and copper/zinc ratio in tissue samples of aortic aneurysms and in the control samples

Mineral component	AA control (*n* = 20) A	AAA (*n* = 47) B	TA control (*n* = 20) C	TAA (*n* = 61) D
	Average ± SD (min.-max.) median (Q1, Q3)			
Cu (μg/g)	1.75 ± 0.69 (0.94–3.03) 1.73 (1.20, 2.11)	2.15 ± 1.39 (0.82–5.81) 1.46 (1.11, 2.66)	1.19 ± 0.25 (0.59–1.56) 1.20 (1.04, 1.40)	1.04 ± 0.32 ^#^ (0.54–2.53) 1.01 (0.82, 1.15)
Zn (μg/g)	29.9 ± 18.88 (8.28–73.35) 22.85 (15.16, 40.06)	18.54 ± 12.3* (1.02–51.22) 14.05 (10.07, 25.67)	14.71 ± 2.64 (10.82–18.34) 15.37 (11.58, 17.23)	12.9 ± 4.05 * (5.34–26.29) 12.48 (10,28, 14.68)
Cu/Zn molar ratio	0.073 ± 0.034 (0.029–0.166) 0.067 (0.058, 0.070)	0.180 ± 0.227 ^ (0.038–1.084) 0.110 (0.078, 0.141)	0.085 ± 0.023 (0.045–0.128) 0.086 (0.071, 0.099)	0.085 ± 0.019 (0.031–0.138) 0.084 (0.072, 0.094)
Se (ng/g)	183.65 ± 84.09 (94.56–397.55) 190,12 (125.9, 210.1)	222.63 ± 108.42 (67.93–375.35) 195.61 (126.5, 347.5)	234.14 ± 32.32 (163.11–286.61) 231.42 (215.9, 256.1)	216.60 ± 64.23 * (108.76–380.59) 206.98 (172.8, 242.0)
Cd (ng/g)	71.01 ± 26.28 (15.31–98.20) 70.54 (62.72, 90.79)	60.06 ± 32.26 (21.24–140.73) 51.87 (32.18, 82.95)	68.66 ± 28.88 (21.15–110.81) 69.64 (49.36, 90.85)	62.45 ± 40.72 (11.12–188.42) 50.01 (34.37, 80.26)
Pb (ng/g)	208.41 ± 205.23 (36.82–678.71) 105.29 (66.42, 309.0)	205.03 ± 202.73 (20.85–870.98) 149.45 (92.76, 247.4)	251.91 ± 126.76 (83.84–498.29) 211.99 (166.7, 321.1)	366.55 ± 238.20 * (11.81–950.25) 303.1 (200.12, 470.8)
*p* value	*p_A/B_ < 0.05 ^*p*_A/B_ < 0.005		*p_C/D_ < 0.05 #*p*_C/D_ < 0.02	

*AA* abdominal aorta, *AAA* abdominal aortic aneurysm, *TA* thoracic aorta, *TAA* thoracic aortic aneurysm, *SD* standard deviation, *Q1* lower quartile, *Q3* upper quartile, *p* level of significance

^*,#,^^ differences between control and aortic aneurysm tissues

The mean concentration of Zn in the aortic wall of patients with TAA and AAA (12.9 ± 4.05 μg/g, 18.54 ± 12.3 μg/g, respectively) was significantly lower (*p* < 0.05) than in the control group samples: 14.71 ± 2.64 μg/g, 29.9 ± 18.88 μg/g, respectively (Table 2).

We observed a significant correlation between concentration of Cu and Zn in the arterial wall samples of patients with TAA and AAA (*r* = 0.601, *p* < 0.005; *r* = 0.477, *p* < 0.05, respectively). We did not found significant correlations between other determined elements. Data are not presented in tables.

Cu/Zn ratio was significantly higher in AAA patients than in control group.

The concentration of Se was significantly decreased in TAA patients compared with the control group (216.6 ± 64.23 ng/g vs. 234.14 ± 32.32 ng/g) (Table 2).

The average size of aneurysm in AAA patients was 73.8 ± 18.5 mm and in TAA patients: 53.4 ± 8.6 mm. We did not observe any correlations between the size of aneurysms and the concentration of examined microelements.

### The Concentration of Toxic Elements

The content of Pb was increased (*p* < 0.05) in TAA patients (366.55 ± 238.2 ng/g vs. 251.91 ± 126.76 ng in the control samples). The concentration of Cd in aneurysmal wall of both types of aneurysm was similar to normal aortas (Table 2).

We did not find correlations between content of toxic elements and the size of aneurysms.

### Molar Ratio of Antioxidant Micronutrients to Toxic Elements

**Fig. 1:**
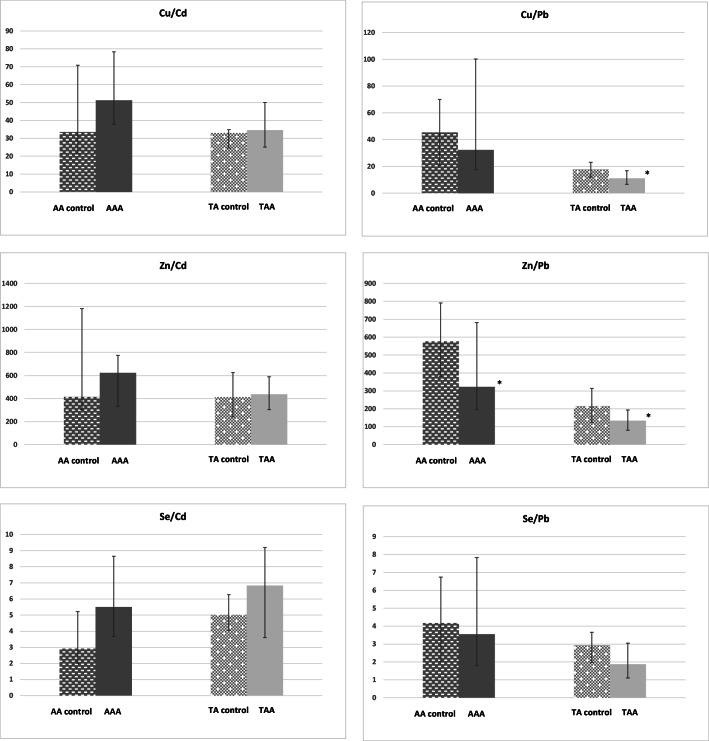
Molar ratio between antioxidant components and toxic elements in tissue samples of aortic aneurysms and in the control samples. Median values with lower and upper quartiles, AA–abdominal aorta, AAA–abdominal aortic aneurysm, TA–thoracic aorta, TAA–thoracic aortic aneurysm, *p < 0.05

We observed significantly (*p* < 0.05) lower median Zn/Pb ratio in both types of aneurysms (AAA: 321.9, Q1–Q3: 196.1–681.7; TAA: 133.1, Q1–Q3: 80.1–192.3) comparing with control samples (AA: 576.7, Q1–Q3: 380.6–791.5; TA: 215.6, Q1–Q3: 125.5–313.6). The median Cu/Pb ratio was significantly lower in TAA patients (11.05, Q1–Q3: 6.69–16.76) than in control group (17.73, Q1–Q3: 12.06–23.04). Data are shown on Fig. 1. Additionally in patients who declared frequent alcohol consumption (vodka), the median Cu/Pb ratio was significantly lower (*p* < 0.05) compared with non-drinkers (7.5, Q1–Q3: 4.5–11.2 vs. 12.0, Q1–Q3: 8.1–19.2). Smoking cigarettes did not affect the molar ratios between the antioxidant micronutrients and toxic elements in both types of aneurysms. Data are not shown on figure.

## Discussion

Imbalance in the concentration of mineral components in the human body is regarded as one of the risk factors for cardiovascular diseases. Our results showed that in patients with TAA, the concentration of Cu in aortic samples is significantly lower, but in AAA patients, it is comparable with normal aorta. Hunter et al. stated elevated concentration of Cu in AA tissue samples, especially in hypertensive patients [26]. The biochemical mechanisms by which hypertension accelerates atherosclerosis and increases the risk of rupture AA are poorly understood. AA patients with hypertension had significantly lower zinc levels in aorta samples. Also, Cu/Zn-superoxide dismutase activity was similarly reduced in the same group of patients. Lower amounts of collagen and elastin in aortas of patients with hypertension were also shown. The authors suggested that the reduction of collagen and elastin in aorta of hypertensive patients with AA compared with their normotensive counterparts may explain the larger size of aneurysms and predispose to eventual rupture. In addition, the diminished antioxidant status associated with hypertension predisposes to lipid peroxidation, which contributes to the acceleration of atherosclerosis processes and risk of rupture AA [26].

Iskra et al. suggested that patients with AAA have deficiency of Cu in the tissue [22]. Studies of Senapati et al. were not consistent with this observation but showed a lower concentration of Zn in comparison with normal aortic wall samples. These studies were limited to a small number of examined patients [27]. In our study, the concentration of Zn was significantly lower in both types of aneurysm compared with normal aortas. Chronic inflammation and degradation of elastin are the main processes in the development of AAA. Recent studies show that Zn has an anti-inflammatory effect [28]. Based on these, diet supplementation with Zn may prove to be an effective adjuvant therapy for the treatment of the AAA. Zinc finger protein A20, a zinc-finger transactivating factor, was identified as a primary response gene following inflammatory stimulation of human umbilical vein endothelial cells [29]. A20 can also be induced in smooth muscle cells and exhibit an anti-inflammatory impact by blockade of nuclear factor κB (NF-κB) signaling [30, 31]. NF-κB can promote chronic inflammation in the aortic wall [32] and regulate MMP transcription [33]. In human and animal experiments, inhibition of NF-κB activation can prevent the development of AAA [34, 35]. Yan et al. studied the effects of Zn on AAA progression and its related molecular mechanism. The results showed that Zn supplementation significantly suppressed the abdominal aortic diameter, as well as a preserved medial elastin fibers in the aortas. Zn supplementation also attenuated infiltration of the macrophages and lymphocytes in the aortas. Furthermore, Zn reduced MMP-2 and MMP-9 production in the aortas and significantly induced A20 protein expression by inhibiting inflammation, along with inhibition of the NF-κB canonical signaling pathway in vitro in aortic vascular smooth muscle cells and in vivo in rat AAA. This study showed that Zn supplementation could prevent the development of rat experimental AAA [36]. However, studies of Ziaja et al. have shown that intraluminal thrombus thickness is not associated with a lower concentration of trace elements in the wall of infrarenal AAA [37]. Koksal et al. compared the concentration of Cu and Zn in tissues of patients with AAA and aortic occlusive disease (AOD). The comparison of tissue Zn levels showed no significant difference. Tissue levels of Cu and thiobarbituric acid reactive substances levels, as a marker of lipid peroxidation, were found to be higher in the AAA group, compared with the AOD group. These results suggest that higher oxidative stress in the AAA, compared with AOD, may be one of the factors contributing to the formation of aneurysms as a result of promoted erosion of the walls [23].

Oxidative stress has been suggested to play a key role in the pathogenesis of AAA [20, 21]. In our study Cu/Zn ratio was significantly higher only in AAA patients in comparison with the control group which indicates a greater role of oxidative stress thusly indicating inflammatory process in this type of aneurysm, than in TAA. Edvinsson et al. examined trace element changes in thoracic aortic dissection. They showed decreased concentrations of Zn and Cu in tissue compared with the control samples. The Cu/Zn ratio in the serum, a marker of infection/inflammation, did not change among patients. These data did not support the hypothesis that inflammation is involved in the pathogenesis of thoracic aortic dissection [25]. Studies of Pincemail et al. showed a significant decrease of plasma levels of Zn and Se, as AAA size increased. Also, Cu/Zn ratio, which is considered as a specific marker of lipid peroxidation, increased, when the size of AAA was higher [38]. In our research, we did not find any correlations between the concentration of the examined elements and the size of aneurysms.

The tissue injury following ischemia-reperfusion is mediated in part by free oxygen radicals. However, studies of Watters et al. suggested that perioperative supplementation with micronutrients with antioxidant properties, such as Zn and Se, has limited effects on strength and physical function after abdominal aortic aneurysmectomy [39]. We showed that the concentration of Se in aortic samples was significantly lower only in TAA patients. An inadequate Se level is supposed to be a risk factor for cardiovascular diseases. European soil, particularly in Central Europe, is relatively low in this micronutrient, which results in low Se content in food [40]. The decreased plasma Se levels have been associated in European populations with development of different cardiovascular diseases, including AAA in the Polish population. In addition to nutritional Se deficiency, cigarette smoking, the most important risk factor for AAA, may substantially contribute to the reduction of Se levels in patients [41]. Studies of Strauss et al. showed the potential role of the selenoprotein P in pathogenesis of AAA. This study provided preliminary genetic evidence that the SEPP1 functional variants contribute to the AAA risk. The results suggest that the rs3877899A allele may increase the susceptibility or promote AAA progression through influencing the risk of peripheral atherosclerosis and isolated systolic hypertension. The SEPP1 rs3877899G-rs7579G haplotype seems to be a factor that increases predisposition to AAA in overweight and obese patients and a potential marker of aggressive-growing AAAs [42]. It should be remembered that in the case of Se, the safety margin between the recommended and the toxic dose is narrow; therefore, supplementation should be preceded by a biochemical examination [43].

We observed significantly higher concentration of Pb in aneurysmal wall of TAA patients. Additionally, we found disturbed proportions between Cu and Pb in TAA and Zn and Pb in both types of aneurysm, while concentration of Cd is similar to normal aortas in both types of aneurysm. Other studies have shown that the elevated levels of Cd in the walls of AAA coexisting with iliac artery aneurysms may suggest an impact on the accumulation of this toxic element and greater damage of the iliac artery wall [24].

## Conclusions

In the examined aneurysms, we have shown the differences in concentrations of mineral components compared with the control tissues. The Zn concentration was decreased in both AAA and TAA samples. Impaired ratio of Zn to Pb may predispose to aortic aneurysms. Further work is required to confirm obtained findings.
